# Linked versus unlinked hospital discharge data on hip fractures for estimating incidence and comorbidity profiles

**DOI:** 10.1186/1471-2288-12-113

**Published:** 2012-08-01

**Authors:** Trang Vu, Lesley Day, Caroline F Finch

**Affiliations:** 1 Injury Research Institute, Monash University, Melbourne, Victoria, 3800, Australia

**Keywords:** Comorbidity, Hip fracture, Incidence, Hospital discharge data, Sensitivity and specificity

## Abstract

**Background:**

Studies comparing internally linked (person–identifying) and unlinked (episodes of care) hospital discharge data (HDD) on hip fractures have mainly focused on incidence overestimation by unlinked HDD, but little is known about the impact of overestimation on patient profiles such as comorbidity estimates. In view of the continuing use of unlinked HDD in hip fracture research and the desire to apply research results to hip fracture prevention, we concurrently assessed the accuracy of both incidence and comorbidity estimates derived from unlinked HDD compared to those estimated from internally linked HDD.

**Methods:**

We analysed unlinked and internally linked HDD between 01 July 2005 and 30 June 2008, inclusive, from Victoria, Australia to estimate the incidence of hospital admission for fall-related hip fracture in community-dwelling older people aged 65+ years and determine the prevalence of comorbidity in patients. Community-dwelling status was defined as living in private residence, supported residential facilities or special accommodation but not in nursing homes. We defined internally linked HDD as the reference standard and calculated measures of accuracy of fall-related hip fracture incidence by unlinked HDD using standard definitions. The extent to which comorbidity prevalence estimates by unlinked HDD differed from those by the reference standard was assessed in absolute terms.

**Results:**

The sensitivity and specificity of a standard approach for estimating fall-related hip fracture incidence using unlinked HDD (i.e. omitting records of in-hospital deaths, inter-hospital transfers and readmissions within 30 days of discharge) were 94.4% and 97.5%, respectively. The standard approach and its variants underestimated the prevalence of some comorbidities and altered their ranking. The use of more stringent selection criteria led to major improvements in all measures of accuracy as well as overall and specific comorbidity estimates.

**Conclusions:**

This study strongly supports the use of linked rather than unlinked HDD in injury research. In health systems where linked HDD are unavailable, current approaches for identifying incident hip fractures may be enhanced by incorporating additional evidence-based criteria.

## Background

Hip fractures are a major injury burden in community-dwelling older people. About 90% of hip fractures occur in people aged 65+ years, with most of these being fall-related and involving community dwellers [1,2]. Initial hospitalisation costs are reportedly high [3-6] and costs of rehabilitation, home nursing, home help and institutional care can be substantial depending on subsequent functional impairment [6,7]. Patients with a hip fracture have been found to have a higher risk of dying than would be expected up to 20 years following the hip fracture [8,9].

Accurate estimates of hip fracture incidence, as well as reliable descriptions of the demographic, comorbidity and risk factor profiles of patients, are required for hip fracture management and prevention [10]. Hospital discharge data (HDD) are appropriate for this purpose because virtually all hip fractures necessitate hospital admission [11]. In health systems where a unique system-wide patient identifier (UPI) is lacking, hospital records refer to specific episodes of care rather than to cases or persons (hereafter referred to as unlinked HDD). To minimise multiple counting, a standard approach for identifying incident hip fractures from unlinked HDD excludes inter-hospital transfers and/or readmissions within 28 or 30 days of discharge [12,13]. Records showing the discharge status as in-hospital death may also be excluded to further minimise multiple counting based on an assumption that patients who die in hospital would have previous hospital admission(s) [14].

The level of incidence overestimation by unlinked HDD has been estimated to be between 7%–31% by one study which directly compared unlinked HDD with person-identifying (linked) HDD [10]. This overestimation, in turn, would be expected to have a direct impact on the accuracy of patient profiles such as comorbidity, but, to date, little is known about this impact. In view of the continuing use of unlinked HDD in hip fracture epidemiological research [3,5,15], and the desire to apply research results to hip fracture prevention, these data should be assessed for potential inaccuracies in both incidence and comorbidity estimates. The aim of this study was therefore to compare unlinked and linked fall-related HDD to determine the sensitivity, specificity, and negative and positive predictive values of unlinked HDD for estimating fall-related hip fracture incidence and determining patients’ comorbidity profiles by using linked HDD as the reference standard. The study focused on community-dwelling older people because the majority of fall-related hip fractures occur in community settings [1].

## Methods

We analysed the Victorian Admitted Episodes Dataset (VAED) between 01 July 2005 and 30 June 2008, inclusive (fiscal years 2005/06, 2006/07 and 2007/08), to estimate the incidence of fall-related hip fracture in community-dwelling people aged 65+ years in Victoria and determine patients’ comorbidity profiles. The VAED is an administrative and clinical data collection of admitted patient episodes in acute hospitals in Victoria, Australia’s second most populous state [16]. This data collection is managed by the Victorian Department of Health (DOH) and used to support casemix funding, epidemiological research, health services planning and policy development [16]. The collection is subject to regular audits which indicate good-to-excellent diagnosis and procedure coding quality [16,17]. The most recent published audits included, among other diagnoses and procedures, Charlson comorbidities, external cause of falls, hip fracture diagnosis and hip replacement [17].

**Figure 1:**
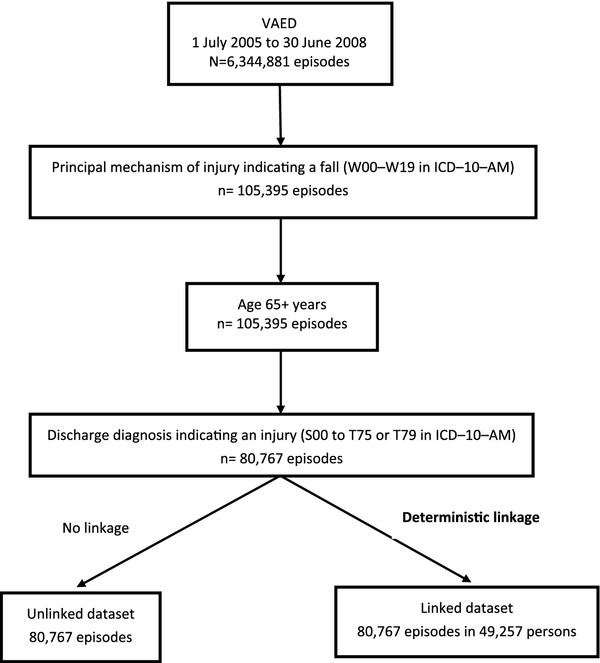
**A flow chart of data extraction process.** ICD–10–AM – International Classification of Diseases, Tenth Revision, Australian Modification. VAED: Victorian Admitted Episode Dataset.

Each patient within a hospital is identified by a unique, hospital generated patient identifier and each episode has a unique hospital derived episode number; however, the VAED lacks a system-wide UPI and does not capture date of injury information [16,18]. Episodes containing the principal mechanism of injury indicating a fall (W00–W19 in the International Classification of Diseases, Tenth Revision, Australian Modification (ICD-10-AM)) [19,20], the age at admission of 65+ years and the principle diagnosis indicating an injury (S00 to T75 or T79 in ICD-10-AM) [19,20] were extracted from the VAED to form an unlinked dataset. The S00 to T75 or T79 range was specified in order to exclude injuries due to medical care procedures [21]. Figure 1 summarises the data extraction process. The unlinked dataset was internally linked by the DOH using stepwise deterministic linkage and person-identifying variables (such as sex, date of birth, country of birth, postcode, and Medicare number and suffix) to produce a linked dataset for the present study [22]. The linkage process and linkage quality have been described in detail elsewhere [22]. Briefly, this process consists of nine steps, including standardisation of linkage variables, determination of the quality of coding and quality assessment of linked data [22]. A DOH study on the quality of VAED internal linkage for the period 1995–2000 found that the quality of coding was high and the false positive rate, defined as the rate of incorrectly matched records, was low (between 1% to 2%) [22]. However, the report indicated that the false negative rate, measured as the percentage of unmatched inter-hospital transfer records from the same patients, was high (15%). A more recent assessment of the quality of VAED internal linkage is not publicly available.

Within the unlinked dataset we used a standard approach of identifying incident fall-related hip fractures [14] (hereafter referred to as the base case) (Table 1)—records were selected if the principal diagnosis was hip fracture (S72.0 to S72.2 in ICD–10–AM [19,20]), the admission source was coded as “private residence/accommodation” and the discharge status was other than in-hospital death. The category “private residence/accommodation” includes people living in their own home or private accommodation and excludes residents in nursing homes [16]. We excluded records indicating readmission within 30 days of discharge; however, given the lack of a UPI in the VAED and the lack of a hospital site identifier for private hospitals this was only possible for patients admitted to the same public hospitals during the study period (64.7% of patients according to the linked dataset). Patients admitted to the same public hospitals during the study period were significantly younger (median age 76 years; interquartile range ((IQR) 60–84) than those admitted to different public hospitals during the study period (median age 81 years; IQR 72–86) (nonparametric equality-of-medians test p <0.001).

**Table 1:** Selection criteria for linked and unlinked Victorian Admitted Episodes Dataset

	**Reference standard**	**Base case**	**S1**	**S2**	**S3**	**S4**
***Inclusion criteria***						
Principal diagnosis hip fracture^*^	✓	✓	✓	✓	✓	✓
Admission source coded as “private residence/accommodation”	✓	✓	✓	✓	✓	✓
***Exclusion criteria***						
Readmission within 30 days of discharge	✓	✓	✓	✓	✓	✓
Readmission within 120 days of discharge	✓			✓	✓	✓
Discharge status denoting in-hospital death		✓		✓	✓	
Hip revision procedures only	✓				✓	✓
Non-acute care type	✓				✓	✓
Non-emergency admission	✓				✓	✓

S1 – Scenario 1. S2 – Scenario 2. S3 – Scenario 3. S4 – Scenario 4.

* S72.0–S72.2 in International Classification of Diseases, Tenth Revision, Australian Modification.

For the linked dataset, we used the same principal diagnosis range and admission source category as those for unlinked data, but disregarded discharge status. We further refined our identification by including only records showing emergency hospital admission for acute care with no hip revision procedure code(s) (Table 1) [15]. Due to the lack of date of injury in the VAED [18], the lack of ICD–10–AM codes on laterality of fractures (Saad P. Disease classification developer, National Centre for Classification in Health (Australia). Personal communication. 30 April 2010) and the inaccuracy of fracture type classification [23], we developed additional criteria to distinguish between the first and subsequent fall-related hip fracture in the same patients. We assumed, based on a literature review, that the minimum time gap (clearance period) between incident fall-related hip fractures in the same patient would be 120 days [24,25] and that all principal external cause codes (mechanism of injury, place of occurrence and activity being undertaken when injured) would differ between different fall-related hip fractures in the same patient. We also performed the analysis without the criterion for external cause codes; however, we found only seven cases that would be identified as incident cases if we omitted this criterion.

We defined the method of identifying incident fall-related hip fracture from linked data as the reference standard and calculated the sensitivity, specificity, positive predictive value (PPV) and negative predictive value (NPV) for the base case using standard definitions [26]. We conducted a sensitivity analysis of the base case under four scenarios (Table 1)— S1 included records showing the discharge status as in-hospital death; S2 excluded records indicating readmission within 120 days of discharge; S3 excluded records indicating readmissions within 120 days of discharge, records with only hip revision procedure code(s), and those with the care type coded as non-acute and the category of admission coded as non-emergency; S4: was the same as S3 but also included records showing the discharge status as in-hospital death (i.e. S4 employed the same selection criteria as the reference standard).

We calculated age-specific hospital admission rates for fall-related hip fracture in community-dwelling people aged 65+ years using Victorian population estimates for relevant years [27-30]. The denominator for each age group was obtained by subtracting the number of residents in nursing homes from the population estimate for this group. We directly standardised rates of hospitalisation to the 2001 Australian standard populations [31].

Patients’ comorbidities were classified using the Deyo adaptation of the Charlson Comorbidity Index (CCI) because this index was constructed using administrative data similar to those collected for the VAED, and validated using the VAED [32]. We also estimated the prevalence of other risk factors for falling and fall-related fractures, including osteoporosis, Parkinson’s disease, visual impairment, deafness and delirium, using ICD–10–AM codes also tested on the VAED [33]. We distinguished comorbidities from adverse events that arose during hospitalisation by utilising a condition-onset flag available in the VAED [16]. The extent to which comorbidity prevalence estimates by unlinked data differed from those by linked data was assessed in absolute terms by performing pairwise comparisons. For patients in the linked dataset with more than one hospitalisation for a fall-related hip fracture, we optimised comorbidity ascertainment by defining the first multiday record as the index hospitalisation and searching this record as well as looking back at previous record(s) for the presence of comorbidities (hereafter referred to as lookback) [34]. Comorbidity was deemed to be present if it was coded in one or more of these records. The median period of lookback was 565 days (IQR 274–836).

The Monash University Human Research Ethics Committee granted approval for this study. We conducted all analyses in Stata version 10 [35]. We evaluated equality of proportions using two-sample chi square tests of proportions or Fisher's exact test, as appropriate. For skewed continuous variables, we compared medians using nonparametric K-sample tests on the equality of medians [35]. A multivariable Poisson regression model controlling for age and sex was used to assess the existence of a trend in fall-related hip fracture hospitalisation rates over time. All tests were two tailed. The level of significance was 5%.

## Results

A total of 10,110 incident fall-related hip fractures in 9,879 community-dwelling older persons were identified from the linked dataset (Table 2). Of these patients, 2.3% had a second fall-related hip fracture; the median time from the first to second fall-related hip fracture admission was 335 days (IQR 216–551 days). No patients had more than two fall-related hip fractures.

**Table 2:** Sensitivity, specificity, positive and negative predictive values of unlinked data in identifying incident fall-related hip fractures

	**Linked data**	**Unlinked data**	**Unlinked data**	**Unlinked data**	**Unlinked data**	**Unlinked data**
	**Ref standard**	**Base case**	**Scenario 1**	**Scenario 2**	**Scenario 3**	**Scenario 4**
No hip fractures	N=10,110	N=11,110	N=11,746	N=11,056	N=10,173	N=10,765
Overestimation	NA	9.9%	16.2%	9.4%	0.6%	6.5%
Sensitivity		94.4% (94.0–94.9)	99.8% (99.7–99.9)	94.4% (93.9–94.8)	94.4% (93.9–94.8)	99.7% (99.6–99.8)
Specificity		97.5% (97.4–97.6)	97.4% (97.2–97.5)	97.6% (97.5–97.7)	99.0% (98.9–99.1)	98.9% (98.8–99.0)
PPV		85.9% (85.3–86.6)	85.9% (85.2–86.5)	86.3% (85.7–87.0)	93.8% (93.3–94.3)	93.7% (93.2–94.1)
NPV		99.1% (99.0–99.2)	100.0% (99.9–100.0)	99.0% (99.0–99.2)	99.1% (99.0–99.2)	100.0% (99.9–100.0)

Note values are percentages (95% confidence intervals) unless stated otherwise. True negatives were community-dwelling Victorian population aged 65+ years hospitalised for fall-related injury other than a hip fracture between 2005/06 and 2007/08.

NA – Not applicable. PPV – Positive predictive value. NPV – Negative predictive value. CI – confidence intervals.

Overall, the base case performed less well compared with the reference standard and the other scenarios tested (Table 2). The inclusion of hospital records showing the discharge status as in-hospital death (S1) produced the highest sensitivity (99.8%) and NPV (100.0%) while maintaining specificity and PPV. Extending the clearance period from 30 to 120 days (S2) marginally reduced the number of cases and improved PPV slightly. The application of additional selection criteria to S2 (exclusion of records showing hip revision procedure(s) only and records having the care type coded as non-acute or having the admission category coded as non-emergency) to create S3 resulted in the highest specificity (99.0%) and PPV (93.8%) while maintaining sensitivity and NPV. Using selection criteria identical to those in the reference standard improved all measures of accuracy in S4 compared with the base case. We found S4 to be more sensitive than S3 while being similar in most other accuracy measures. Scenario S3 was as sensitive as S1.

The age-adjusted fall-related hip fracture hospital admission rate per 100,000 population dropped from 520.6 in 2005/06 to 511.2 in 2006/07 and 492.6 in 2007/08 according to the linked dataset. These downward trends over time were not consistently statistically significant (p >0.05 for the decline between 2005/06 and 2006/07, and p <0.05 for the decline between 2005/06 and 2007/08 and between 2006/07 and 2007/08). However, we observed a statistically significant trend toward higher hospital admission rates in older age groups in both men and women (all p <0.001). Women outnumbered men in every age category.

Although age-specific and age adjusted/standardised hospital admission rates differed across all methods, gender distributions did not vary significantly between datasets, rates in the 65–69 and 70–74 age groups were broadly similar between datasets, and the statistically inconsistent downward trends across time observed in the linked data was also seen with the unlinked data. The median age at admission, sex distribution, marital status and Indigenous status were also similar across datasets. In Australia, the Indigenous status of patients is obtained through self-identification. An assessment of the accuracy of Indigenous status in Victorian HDD for 2001/02 reported a 22% underestimation rate [36].

According to the linked data the prevalence of any Charlson comorbidity was 31.1% and the prevalence of any Charlson or non-Charlson comorbidity was 37.3% (Table 3). The most common comorbid conditions among patients were renal disease (7.6%), dementia (7.1%), congestive heart failure (5.7%), diabetes (5.4%), diabetes with complications (5.3%), and pulmonary disease (4.3%). The least prevalent comorbidities were neuromyalgia (<5 cases), liver disease (mild, moderate and severe 0.2% in total), peptic ulcer (0.2%), ataxia (0.1%) and human immunodeficiency virus (0.0%).

**Table 3:** Comorbidities in community-dwelling older people hospitalised for fall-related hip fracture by data source

**Comorbidity**	**Linked data ** ** Ref standard**	**Unlinked data** ** Base case**	**Unlinked data** ** Scenario 1**	**Unlinked data** ** Scenario 3**	**Unlinked data** ** Scenario 4**
Any comorbidity	37.3 (36.3–38.2)	30.7 (29.8– 31.5)	32.0 (31.2–32.9)	31.2 (30.3– 32.1)	32.6 (31.7– 33.5)
Diabetes	5.4 (4.9–5.8)	4.4 (4.0–4.8)	4.5 (4.1–4.9)	4.5 (4.1–4.9)	4.5 (4.1–4.9)
Diabetes complications	5.3 (4.8–5.7)	4.2 (3.8 –4.6)	4.4 (4.1–4.8)	4.3 (3.9–4.7)	4.5 (4.1–4.9)
Renal disease	7.6 (7.1–8.1)	5.8 (5.4 –6.3)	6.5 (6.1–7.0)	6.0 (5.5–6.5)	6.7 (6.3–7.2)
Dementia	7.1 (6.6–7.6)	5.6 (5.2 –6.1)	5.8 (5.3–6.2)	5.8 (5.4–6.3)	6.0 (5.6–6.5)
Congestive heart failure	5.7 (5.3–6.2)	4.0 (3.6 –4.4)	4.8 (4.4–5.2)	4.1 (3.7–4.5)	4.9 (4.5–5.3)
Pulmonary disease	4.3 (3.9–4.7)	3.2 (2.9 –3.6)	3.6 (3.2–3.9)	3.3 (3.0–3.7)	3.6 (3.3–4.0)
Osteoporosis	3.9 (3.5–4.3)	4.3 (3.9 –4.7)	4.3 (3.9–4.6)	4.4 (4.0–4.8)	4.3 (3.9–4.7)
Parkinson’s disease	3.0 (2.7–3.3)	2.7 (2.5-3.1)	2.7 (2.4–3.0)	2.7 (2.4–3.0)	2.7 (2.4–3.0)
Delirium	2.6 (2.3–2.9)	1.8 (1.6–2.1)	1.9 (1.7–2.2)	1.9 (1.7–2.2)	2.0 (1.8–2.3)
Cerebral vascular accident	2.2 (1.9–2.5)	1.6 (1.4–1.9)	1.6 (1.4–1.9)	1.7 (1.4–1.9)	1.7 (1.4–1.9)
AMI	2.0 (1.7–2.2)	1.3 (1.1–1.5)	1.5 (1.3–1.7)	1.3 (1.1–1.6)	1.5 (1.3 –1.8)
Cancer	1.8 (1.6–2.1)	1.5 (1.3–1.8)	1.6 (1.4– 1.9)	1.5 (1.3–1.7)	1.6 (1.4–1.9)
Vision impairment	1.7 (1.5–2.0)	1.3 (1.1–1.5)	1.3 (1.1– 1.5)	1.3 (1.1–1.6)	1.3 (1.1– 1.5)
Deafness	1.4 (1.2–1.7)	1.0 (0.8–1.2)	1.0 (0.8– 1.2)	1.0 (0.8–1.2)	1.0 (0.9– 1.3)
Paraplegia	1.3 (1.1–1.5)	0.9 (0.8–1.1)	0.9 (0.8– 1.1)	1.0 (0.8–1.2)	1.0 (0.8–1.1)

Note values are percentages (95% confidence intervals) unless stated otherwise. Results for Scenario 2 are identical or almost identical to those in the base case and hence not shown here. Prevalence <1% not presented (metastatic cancer, peripheral vascular disease, peptic ulcer, liver disease, severe liver disease, ataxia, human immunodeficiency virus and connective tissue disorder).

AMI – Acute myocardial infarction.

Trends towards lower comorbidity prevalence estimates by unlinked data were observed; however, differences between these estimates and those by linked data were not consistently statistically significant across all unlinked vs. linked comparisons, except for the prevalence of any comorbidity and the prevalence of dementia (p-values <0.001 and <0.05, respectively). The S4 selection method performed better than the base case and other scenarios in having the least number of comorbidities whose prevalence was significantly different from that estimated by linked data.

The top three comorbidities and their ranking were the same irrespective of the data source and case selection method: these included (in descending order) diabetes (with or without complications), renal disease and dementia. These three comorbidities accounted for approximately 55.0% of comorbidities in the hip fracture patients according to the linked data. Similar estimates (54.3%–55.3%) were obtained from the unlinked data. Nevertheless, the data sources did not agree on the fourth and fifth most common comorbidity. Osteoporosis was identified as either the fourth or fifth most common comorbidity among incident cases by methods using the unlinked data but was not rated in the top five comorbidities by the linked data. The reverse was found for pulmonary disease. Another difference between the linked and unlinked data was that the former showed gender differences in the prevalence of osteoporosis with a female to male ratio of 6.6 (p <0.001) but not the latter (p >0.30 for all methods using the unlinked data).

## Discussion

Methods of estimating fall-related hip fracture incidence in community-dwelling older people using unlinked HDD overestimated fall-related hip fracture incidence, underestimated the prevalence of some co-morbid conditions in patients and altered the relative ranking of the top five comorbidities. To our knowledge, this is the first study to concurrently assess the accuracy of both incidence and comorbidity estimates in the comparison of linked with unlinked HDD. Two similar comparison studies have been conducted but they were only incidence studies; one study examined the incidence of hospitalised injurious falls and results for hip fracture were not reported separately [12] whereas the other study focused on hip fractures and our estimates of incidence overestimation by the base case and its variants are within the range of estimates reported by this study [10]. Our results support earlier recommendations that linked rather than unlinked HDD be used in injury incidence estimation and provide further rationale for these recommendations by describing comorbidity differences between these two data sources.

In health systems where linked HDD are unavailable, S4 appears to be the most promising method. The use of the most stringent selection criteria in S4 led to major improvements in all accuracy measures as well as demographic estimates, and overall and specific comorbidity estimates. These criteria would be simple and easy to implement if an unlinked data source has admission and care details similar to those contained in the VAED.

Our estimate of the prevalence of any Charlson comorbidity, and for specific Charlson comorbidities (except dementia and diabetes with or without complications) using linked data is lower than previously reported [9,37]. The underestimation of comorbidity prevalence by linked data in our study may be attributed to the fact that we ascertained patient comorbidity from HDD instead of chart review. It is commonly acknowledged that HDD tend to underestimate comorbidity prevalence [38]. The lower comorbidity estimates in our study may also be explained by the fact that up to 30% of patients included in recent studies [8,9,37] were from nursing homes; these patients would have had a higher comorbidity burden than community-dwelling older people in our study.

Very few published hip fracture studies have reported fall and fracture risk factors in patients alongside Charlson and non-Charlson comorbidities. Our estimate of 3.9% for osteoporosis based on linked data is slightly higher than that based on self-reported data on the Australian adult population (3.0%); however, the female-to-male ratio is similar [39,40]. The discrepancy in the prevalence of osteoporosis may be explained by differences in the age distribution in our study and the adult population. The prevalence of visual impairment in patients identified by the linked data (1.7%) is considerably lower than those reported for older Australians (4.7% in 60–69 years, 11.1% in 70–79 years and 28.7% in 80+ years) [41] suggesting that visual factors may not be routinely assessed in hip fracture patients. Another possible explanation is that visual factors may be assessed routinely but not recorded or coded in HDD.

The annual number and age-standardised rate of fall-related hip fractures per 100,000 population have been proposed for inclusion in a set of fall injury outcome metrics for use when evaluating progress in fall prevention programs for older people [42]. Our study has demonstrated that although incidence estimates based on unlinked HDD (except S3) are biased upward due to multiple counting, they appear to be suitable for monitoring sex differences and trends across time.

The absence of date of injury information in the VAED made it difficult to distinguish multiple admissions for the same fall-related hip fracture from new admissions associated with a recurrent fall-related hip fracture. Our strategy for dealing with this problem was to use a clearance period based on a literature review in conjunction with information from combinations of variables. Our clearance period compares favourably with the observed window of time during which the risk for a recurrent hip fracture is highest [24,25], and is consistent with the timeframe during which the risk of readmission is high [43,44]. Furthermore, our estimate of the incidence of a second fall-related hip fracture is consistent with estimates reported in the literature [45]. Our evidence-based criteria for identifying incident fall-related hip fractures from linked HDD in the absence of the date of injury were recently validated against a gold standard which used the date of injury and found to be highly accurate (unpublished study).

Our study, however, has some limitation which must be acknowledged. The category of patients in the VAED used to represent community-dwelling people includes people from prisons, armed forces base camps/hospitals, supported residential facilities (excluding nursing homes) and special accommodation houses. Some of the patients included in our datasets were likely to have resided in one of these facilities prior to hospitalisation; however, the VAED does not contain supplemental information to complement descriptions of accommodation categories. Nevertheless, this classification error is non-differential; therefore, it should not impact on our study findings.

Finally, the lookback study we conducted was not comprehensive because we only had access to patients’ previous hospitalisation records if these were fall-related. In addition, episodes of care that started or concluded at the beginning of the study period would have had no prior records in our dataset. This could be another contributing factor to the lower comorbidity estimates based on linked data in our study.

## Conclusions

Methods of estimating fall-related hip fracture incidence in community-dwelling older people using unlinked HDD overestimate fall-related hip fracture incidence, underestimate the prevalence of some comorbid conditions and may alter the relative ranking of these conditions. These results re-enforce earlier recommendations that linked rather than unlinked HDD be used in injury research. The utility of linked HDD would be enhanced by the inclusion of the date of injury. In health systems where linked HDD are unavailable, current approaches for identifying incident hip fractures may be enhanced by incorporating additional evidence-based criteria.

## Competing interests

The authors declared that they have no competing interest.

## Authors’ contributions

TV conceived and planned the study, applied for ethics approval, obtained hospitalisation data, defined and performed the statistical analysis, and drafted the manuscript and coordinated the contribution from CFF and LD. CFF and LD contributed to the conception, interpretation of data and critical appraisal of the manuscript. All authors read and approved the final manuscript.

## References

[B1] Kreisfeld R Newson R Hip fracture injuries 2006 AIHW National Injury Surveillance Unit, In. Adelaide

[B2] Leslie WD O’Donnell S Jean S Lagace C Walsh P Bancej C Morin S Hanley DA Papaioannou A Trends in hip fracture rates in Canada JAMA 2009 302 8 883 889 19706862 10.1001/jama.2009.1231

[B3] Australian Institute of Health and Welfare (AIHW) The problem of osteoporotic hip fracture in Australia. Bulletin no. 76. Cat. no. AUS 121 2010 AIHW, Canberra

[B4] Lawrence TM White CT Wenn R Moran CG The current hospital costs of treating hip fractures Injury 2005 36 1 88 91 15589923 10.1016/j.injury.2004.06.015

[B5] Gehlbach SH Avrunin JS Puleo E Trends in hospital care for hip fractures OsteoporosInt 2007 18 5 585 591 10.1007/s00198-006-0281-0 17146592

[B6] Haentjens P Autier P Barette M Boonen S The economic cost of hip fractures among elderly women. A one-year, prospective, observational cohort study with matched-pair analysis. Belgian Hip Fracture Study Group J Bone Joint Surg Am 2001 83-A 4 493 500 11315777

[B7] Tiedemann AC Murray SM Munro B Lord SR Hospital and non-hospital costs for fall-related injury in community-dwelling older people NSW Public Health Bull 2008 19 10 161 165 10.1071/nb07022 19091181

[B8] Kannegaard PN van der Mark S Eiken P Abrahamsen B Excess mortality in men compared with women following a hip fracture. National analysis of comedications, comorbidity and survival Age Ageing 2010 39 2 203 209 20075035 10.1093/ageing/afp221

[B9] Vestergaard P Rejnmark L Mosekilde L Increased mortality in patients with a hip fracture-Effect of pre-morbid conditions and post-fracture complications OsteoporosInt 2007 18 12 1583 1593 10.1007/s00198-007-0403-3 17566814

[B10] Brophy S John G Evans E Lyons R Methodological issues in the identification of hip fractures using routine hospital data: A database study OsteoporosInt 2006 17 3 405 409 10.1007/s00198-005-2038-6 16308674

[B11] Boufous S Finch C Close J Day L Lord S Hospital admissions following presentations to emergency departments for a fracture in older people InjPrev 2007 13 3 211 214 10.1136/ip.2006.014654 PMC2598384 17567981

[B12] Boufous S Finch C Estimating the incidence of hospitalized injurious falls: Impact of varying case definitions InjPrev 2005 11 6 334 336 10.1136/ip.2005.009837 PMC1730302 16326765

[B13] Clark DE DeLorenzo MA Lucas FL Wennberg DE Epidemiology and short-term outcomes of injured medicare patients J Am GeriatrSoc 2004 52 12 2023 2030 10.1111/j.1532-5415.2004.52560.x 15571537

[B14] Cassell E Clapperton A A decreasing trend in fall-related hip fracture incidence in Victoria, Australia Osteoporos Int 2012 10.1007/s00198-012-1937-6 22349962

[B15] Dodds MK Codd MB Looney A Mulhall KJ Incidence of hip fracture in the Republic of Ireland and future projections: A population-based study OsteoporosInt 2009 20 12 2105 2110 10.1007/s00198-009-0922-1 19337676

[B16] Department of Human Services (DHS) VAED Manual 2008 18 DHS, In. Melbourne

[B17] Henderson T Shepheard J Sundararajan V Quality of diagnosis and procedure coding in ICD-10 administrative data Med Care 2006 44 11 1011 1019 17063133 10.1097/01.mlr.0000228018.48783.34

[B18] Hayen AD Boufous S Harrison JE A discussion of the potential benefits to injury surveillance through inclusion of date of injury in hospitalisation data in New South Wales and Australia NSW Public Health Bull 2007 18 7–8 130 132 10.1071/nb07059 17854542

[B19] National Centre for Classification in Health (NCCH) The International Statistical Classification of Diseases and Related Health Problems, 10th Revision, Australian Modification (ICD-10-AM) Fourth Edition 2004 NCCH, In. Sydney 10169442

[B20] National Centre for Classification in Health (NCCH) The International Statistical Classification of Diseases and Related Health Problems, 10th Revision, Australian Modification (ICD-10-AM) Fifth Edition 2006 NCCH, In. Sydney 10169442

[B21] Bradley C Hospitalisations due to falls by older people, Australia 2008–09. Injury research and statistics series no. 62. Cat. no. INJCAT 138 2012 Australian Institute of Health and Welfare, In. Canberra

[B22] Sundararajan V Henderson T Ackland MJ Marshall R Linkage of the Victorian admitted episodes dataset Symposium on health data linkage: its value for Australian health policy development and policy relevant research 2002 Sydney, Australia Available at http://www.publichealth.gov.au/publications/symposium-on-health-data-linkage:-its-value-for-australian-health-policy-development-and-policy-relevant-research:-proceedings (accessed 15 September 2011)

[B23] Ryg J Rejnmark L Overgaard S Brixen K Vestergaard P Hip fracture patients at risk of second hip fracture: A nationwide population-based cohort study of 169,145 cases during 1977–2001 J Bone Miner Res 2009 24 7 1299 1307 19257816 10.1359/jbmr.090207

[B24] Nymark T Lauritsen JM Ovesen O Rock ND Jeune B Short time-frame from first to second hip fracture in the Funen County Hip Fracture Study OsteoporosInt 2006 17 9 1353 1357 10.1007/s00198-006-0125-y 16823545

[B25] National Health Service (NHS) Quality Improvement Scotland Surgical profiles for Scottish NHS Boards. Criteria 2007 2007 In.: NHS Available at: http://www.indicators.scot.nhs.uk/Surg_Docs/Criteria_2007.doc. Accessed 03 December 2009

[B26] Sackett DL Haynes RB Guatt GH Tugwell P Clinical epidemiology. A basic science for clinical medicine 1991 2 Little, Brown and Company, Boston

[B27] Australian Institute of Health and Welfare (AIHW) Residential aged care in Australia 2006–07: A statistical overview. Aged care statistics series 26. Cat. no. AGE 56 2008 AIHW, Canberra

[B28] Australian Institute of Health and Welfare (AIHW) Residential aged care in Australia 2007–08: A statistical overview. Aged care statistic series 28. Cat. no. AGE 58 2009 AIHW, Canberra

[B29] Australian Bureau of Statistics (ABS) Population by age and sex, Australian States and Territories 2009 ABS, Canberra

[B30] Australian Institute of Health and Welfare (AIHW) Residential aged care in Australia 2005–2006: A statistical overview. Aged care statistics series 24. Cat. no. AGE 54 2007 AIHW, Canberra

[B31] Australian Institute of Health and Welfare (AIHW) Australia’s health 2008 2008 AIHW, Canberra

[B32] Sundararajan V Henderson T Perry C Muggivan A Quan H Ghali WA New ICD-10 version of the Charlson comorbidity index predicted in-hospital mortality J ClinEpidemiol 2004 57 12 1288 1294 10.1016/j.jclinepi.2004.03.012 15617955

[B33] Brand CA Sundararajan V A 10-year cohort study of the burden and risk of in-hospital falls and fractures using routinely collected hospital data QualSaf Health Care 2010 10.1136/qshc.2009.038273 20558479

[B34] Preen DB Holman CDAJ Spilsbury K Semmens JB Brameld KJ Length of comorbidity lookback period affected regression model performance of administrative health data J ClinEpidemiol 2006 59 9 940 946 10.1016/j.jclinepi.2005.12.013 16895817

[B35] StataCorp Stata Statistical Software: Release 11 2009 StataCorp LP, In. College Station, TX

[B36] Australian Institute of Health and Welfare (AIHW) Improving the quality of Indigenous identification in hospital separations data 2005 AIHW, In. Canberra

[B37] Vidal EI Moreira-Filho DC Coeli CM Camargo KR Fukushima FB Blais R Hip fracture in the elderly: Does counting time from fracture to surgery or from hospital admission to surgery matter when studying in-hospital mortality? Osteoporos Int 2009 20 5 723 729 18839050 10.1007/s00198-008-0757-1

[B38] Humphries KH Rankin JM Carere RG Buller CE Kiely FM Spinelli JJ Co-morbidity data in outcomes research: Are clinical data derived from administrative databases a reliable alternative to chart review? J ClinEpidemiol 2000 53 4 343 349 10.1016/s0895-4356(99)00188-2 10785564

[B39] Australian Institute of Health and Welfare (AIHW) Arthritis and osteoporosis in Australia 2008. Arthritis series no. 8. Cat. no. PHE 106 2008 AIHW, Canberra

[B40] Access Economics The economic cost of not adhering to bisphosphonate treatment for osteoporosis 2006 Access Economics, Canberra Available at http://www.accesseconomics.com.au/publicationsreports/showreport.php?id=109&searchfor=2006&searchby=year. Accessed 20 November 2009

[B41] Australian Institute of Health and Welfare (AIHW) Vision problems among older Australians. AIHW bulletin no. 27 2005 AIHW, Canberra Available from http://www.aihw.gov.au/publications/index.cfm/title/10141. Accessed August 12, 2010

[B42] Dowling AM Finch CF Baseline indicators for measuring progress in preventing falls injury in older people Aust N Z J Public Health 2009 33 413 417 19811475 10.1111/j.1753-6405.2009.00421.x

[B43] Boockvar KS Halm EA Litke A Silberzweig SB McLaughlin M Penrod JD Magaziner J Koval K Strauss E Siu AL Hospital readmissions after hospital discharge for hip fracture: Surgical and nonsurgical causes and effect on outcomes J Am GeriatrSoc 2003 51 3 399 403 10.1046/j.1532-5415.2003.51115.x 12588585

[B44] Teixeira A Trinquart L Raphael M Bastianic T Chatellier G Holstein J Outcomes in older patients after surgical treatment for hip fracture: A new approach to characterise the link between readmissions and the surgical stay Age Ageing 2009 38 5 584 589 19596738 10.1093/ageing/afp124

[B45] Weatherall M Contralateral fracture of the proximal femur. Implications for planning trials J Bone Joint Surg Br 1999 81 1 77 79 10068008 10.1302/0301-620x.81b1.8959

